# Stable and Durable Conductive Superhydrophobic Coatings Prepared by Double-Layer Spray Coating Method

**DOI:** 10.3390/nano11061506

**Published:** 2021-06-07

**Authors:** Xiang Liu, Kai Chen, Dekun Zhang, Zhiguang Guo

**Affiliations:** 1 School of Mechatronic Engineering, China University of Mining and Technology, Xuzhou 221116, China; TB17050017B4@cumt.edu.cn; 2 School of Materials and Physics, China University of Mining and Technology, Xuzhou 221116, China; cumtck@cumt.edu.cn; 3 Hubei Collaborative Innovation Center for Advanced Organic Chemical Materials and Ministry of Education Key Laboratory for the Green Preparation and Application of Functional Materials, Hubei University, Wuhan 430062, China; 4 State Key Laboratory of Solid Lubrication, Lanzhou Institute of Chemical Physics, Chinese Academy of Sciences, Lanzhou 730000, China

**Keywords:** superhydrophobic coating, double-layer spray coating, conductivity, wear resistance, durability

## Abstract

Herein, a low cost, durable, and stable conductive superhydrophobic composite coating (CSC coating) was fabricated on a Q345 steel surface by simple double-layer spray coating. The water contact angle (WCA) of the CSC coating was 160° and the sliding angle (SA) was 3°. In addition to its excellent conductivity (3.10 × 10^3^ Ω), the fabricated composite coating had good durability and wear resistance. After 10 sand-washing cycles, the CSC coating surface still exhibited stable superhydrophobicity (149° WCA, 9.5° SA). At 200 g pressure, the surface of the optimized CSC coating still maintained fine superhydrophobicity (150° WCA, 9.2° SA) and conductivity (1.86 × 10^4^ Ω) after 10 abrasion cycles. In addition, it also exhibited fine adhesion (0.307 MPa) between the composite coating and the substrate. This functional superhydrophobic surface can be applied in specialty fields with harsh conditions such as coal mining and petrochemical activities. This new coating may also expand the application fields of superhydrophobic surfaces and have broad practical application prospects.

## 1. Introduction

The complex and challenging working environments of mining operations routinely causes corrosion damage to equipment, thus endangering production and safety [1,2]. In addition, there are flammable and explosive substances such as gas and coal dust in coal mines. If a large amount of static charge accumulates on a coating surface, electric sparks can be easily produced due to the accumulation of static charges, which can cause serious losses in coal mine production [3]. Therefore, the coatings used in mines must have good corrosion resistance as well as excellent anti-static properties. At present, common conductive anti-corrosive coatings, with their poor comprehensive performance, make it difficult to address conductivity and anti-corrosion problems [4,5]. Hence, the key to the development of such coatings is to research and improve their comprehensive performance.

Inspired by natural organisms, superhydrophobic surfaces, defined as surfaces with contact angles greater than 150 degrees and rolling angles less than 10 degrees, have attracted extensive attention from many experts and scholars [6,7,8]. They have been applied in specific fields due to their special properties, which include self-cleaning [9,10,11,12], corrosion-resistant [13,14,15], drag-reducing [16,17], and anti-icing abilities [18,19,20]. Superhydrophobic surfaces have been proven to possess excellent corrosion resistance [21,22]. Coatings with excellent anti-corrosive and conductive properties can be prepared by combining superhydrophobicity and conductivity features, which can improve comprehensive performance and overcome the problems that plague traditional mine coatings in terms of both conductivity and corrosion protection [23]. 

To date, there have been several studies [24,25,26] examining conductive superhydrophobic surfaces. For instance, Liu et al. [24] used carbon nanotubes (CNTs) and carbon black (CB) as conductive fillers and mixed them with methyl cellulose (MC) to prepare a conductive suspension. Then, a conductive superhydrophobic paper was prepared by immersing the printed paper in a CB/CNT/MC suspension and hydrophobic fumed silica (Hf-SiO_2_) suspension in turn. Gao et al. [25] adopted rubber banding (RB) as a base, Ag nanoparticles (AgNPs) as conductive particles, and polydimethylsiloxane (PDMS) with a low surface energy to fabricate a fluorine-free superhydrophobic and conductive rubber composite with outstanding de-icing performance, which could be used for highly sensitive and stretchable strain sensors. Sun et al. [26] obtained healable conductive superhydrophobic films by combining electrothermal and superhydrophobic properties. Nevertheless, the above preparation approaches with complex processes and high costs are difficult to realize in industrial applications. Therefore, fabricating stable, durable, and widely applied superhydrophobic surfaces still remains a great challenge.

Spray coating, a simple coating preparation method, can be widely used in engineering practice and is not limited by matrix materials or shapes [27,28,29]. However, poor adhesion between the coating and the substrate always limits further applications of the spray coating method. Some studies [30,31,32] have shown that spray coating the bottom layer can improve the adhesion between the coating and the substrate. In this study, a durable and stable conductive superhydrophobic surface was obtained by simple double layer-spray coating. First, a polyphenylene sulfide (PPS) dispersion solution was evenly sprayed on the substrate surface with a spray gun to form a bottom layer, which can provide good adhesion to the substrate surface. Then, a top layer dispersion solution containing modified carbon nanofibers (CNFs), CeO_2_, polytetrafluoroethylene (PTFE), PPS, and PDMS was homogeneously sprayed onto the bottom layer. The composite coating was obtained by heat curing. The prepared surface displayed excellent superhydrophobicity and conductivity as well as durability and wear resistance. In addition, it also exhibited fine adhesion between the composite coating and the substrate. Along with mining coating applications, the preparation of this functional superhydrophobic surface can also provide theoretical reference value for aviation, defense, and petrochemical coatings research. This new coating can also expand the application fields of superhydrophobic surfaces and has broad practical application prospects. 

## 2. Experiment

### 2.1. Materials

CNFs, 100 nm in diameter and 20–200 µm in length, were obtained from Sigma-Aldrich Co. Ltd (Saint Louis, MI, USA). To enhance the dispersion of pristine CNFs in solution, sodium dodecylbenzene sulfonate (SDBS), supplied by Macklin (Shanghai, China), was used to modify the carbon nanofibers, and the mass ratio of CNFs:SDBS in 100 mL deionized water was 1:1. Cerous nitrate (Ce(NO_3_)_3_·6H_2_O) was supplied by the Chengdu Kelong Chemical Reagent Factory (Chengdu, China). Urea (CO(NH_2_)_2_, 99.0%) was provided by the Shantou Xilong Chemical Co. Ltd (Shantou, China). Tetrapropylammonium bromide (TPAB) was obtained from the Sinopharm Chemical Reagent Co. Ltd (Shanghai, China). According to our previous work [33], 3D flower-like CeO_2_ particulates were synthesized with a simple procedure. PPS (average Mn: 1 × 10^4^, yellow color powder) was purchased from Sigma-Aldrich Co. Ltd (Saint Louis, MI, USA). PTFE powder (500–600 mesh) was supplied by the Shanghai Weiwei Plasticizing Co. Ltd (Shanghai, China). PDMS was provided by Dow Silicones Corporation in the USA (Midland, MI, USA). All the materials and reagents were used without further analysis and as received from the vendor. 

### 2.2. Preparation of Conductive Superhydrophobic Composite Coating

The conductive superhydrophobic composite coating was obtained by a simple double layer spray coating method. The substrate specimens were first polished with sandpapers and cleaned ultrasonically for 20 min in alternate baths of acetone and ethanol. At room temperature, a certain amount of PPS powder was added into ethanol to form a dispersion solution (0.02 g/mL). Afterward, the prepared dispersion solution was sprayed onto the surface of steel sheets using a N_2_ gas spray gun. Subsequently, the sprayed specimens were heated at 320 °C for 40 min to obtain the bottom layer of the CSC-coating, which served to increase the adhesion between the composite coating and the substrate. 

**Figure 1:**
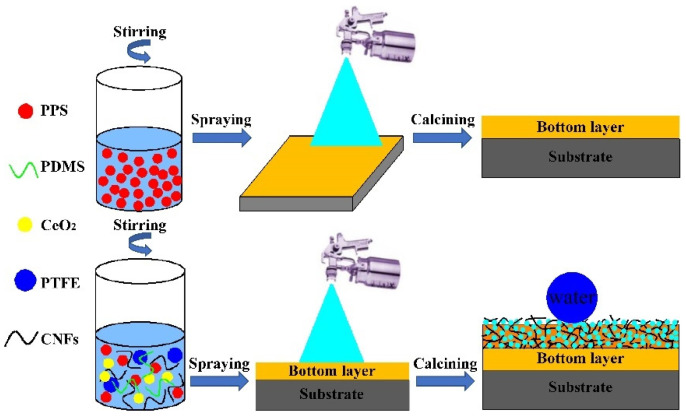
Schematic diagram of the preparation process for conductive superhydrophobic composite coating.

The top layer dispersion solution was prepared by adding a certain amount of the modified CNFs, CeO_2_ particulates, PTFE, 0.4 g PPS, and 0.1 g PDMS into 50 mL absolute ethyl alcohol under ultrasonic agitation for 2 h. Then, the prepared top layer dispersion solution was sprayed onto the bottom layer. Finally, the CSC-coating was obtained by heating the sprayed samples at 330 °C for 2 h. A schematic diagram of the preparation process for the CSC coating is shown in Figure 1.

### 2.3. Durability

Blade scraping and sand washing were used to evaluate the durability of the CSC coating. Specifically, the surface of the CSC coating was scratched by a knife to obtain oblique cross scratches. The sand-washing test was carried out with a self-built device, and 60 g sand fell vertically from a 35 cm height over 50 s, which was recorded as a completion cycle. After blade scraping and sand washing, the durability of the CSC coating was estimated by the change in the contact and sliding angle on the surface. 

### 2.4. Adhesiveness 

The adhesiveness of the CSC coating was measured by grid and pull-off tests. According to GB/T 9286-1998, the grid experiment was completed with a cross-cut tester. Under conditions of uniform pressure and velocity, the surface of the CSC coating was cut by the cross-cut tester. Afterward, the sample was rotated 90 degrees to repeat the above operations, and the surface debris was blown away with a hairdryer. An adhesion tester (F510-20T) was selected to measure the adhesive force of the CSC coating.

### 2.5. Wearability

The wear positive pressure was provided by 200 g weight, and 400 mesh metallographic sandpaper, which was closely bonded to the surface of the CSC coating, was selected as the friction surface. Then, the composite coating samples were slid straight against the sandpaper surface, and the sliding distance was 100 mm. Subsequently, the sample was rotated 90 degrees to repeat the above operations. This reciprocating sliding was recorded as a completed sliding cycle and a total of 20 sliding cycles were carried out for each specimen. 

### 2.6. Characterization

The surface morphologies of the particles and coated specimens were sputtered with Au before testing, and observed by field emission scanning electron microscopy (FESEM, JSM-6701F, Japan Electronics Co., Tokyo, Japan, measured current of 10 µA, accelerating voltage of 5 kV). The water contact angle (WCA) and sliding angle (SA) were measured with 5 µL and 8 µL deionized water droplets, respectively, on a contact angle (CA) system (JC2000D1CA meter, Shanghai Zhongchen digital technology equipment Co. Ltd., Shanghai, China). The average values of WCA and SA were obtained by measuring five different regions of the sample. The element distribution and chemical compositions were analyzed by energy-dispersive x-ray spectroscopy (EDS, JSM-5600LV, Japan Electronics Co., Tokyo, Japan). A resistivity meter (MCP-T610, Mitsubishi, Tokyo, Japan) with a four-point probe was used to measure the electrical conductivity of the samples with different modified CNF contents. The average values of surface resistance were obtained by measuring five different regions of the sample. The bouncing process of droplets on the coating surface was recorded by high-speed camera (VW-9000C, Keyence, Osaka, Japan) where the frame number is 10,000 fps and the shutter speed is 1/30,000 s.

## 3. Results and Discussion

### 3.1. Fabrication of the CSC-Coating

**Figure 2:**
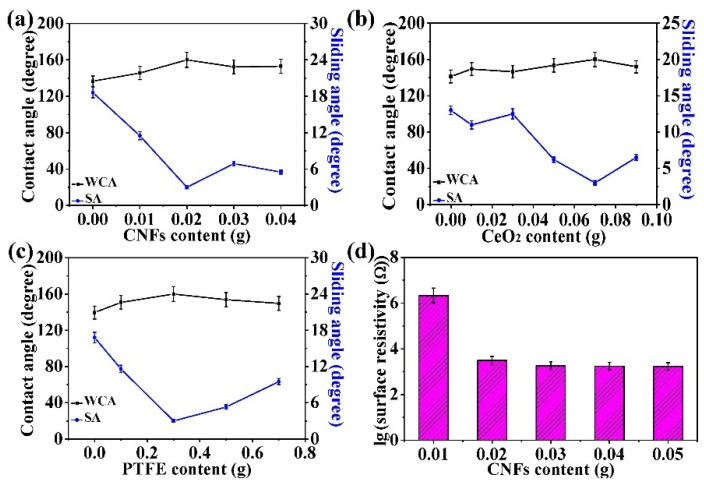
Line charts of the contact angle and sliding angle of the CSC coating at different (**a**) CNFs, (**b**) CeO_2_, and (**c**) PTFE contents. (**d**) Variations in the surface resistivity with CNF content.

Spray coating, which is not limited by the shape and size of parts, is a simple method used to manufacture coatings on various substrates and can be extended in practical applications [34]. From these line charts in Figure 2a–c, it can be easily seen that WCA increase first and then decrease with increasing additive content, which is contrary to the change trend of SA under different additive contents. The above phenomenon can be explained by Wenzel’s theory [35]. The PDMS coated on the surface of the particles provide the low surface energy for the coating. Increasing additives improve the surface roughness of the coatings, which is conducive to enhancing the hydrophobicity of the coating surface. Nevertheless, when the additives are excessive, the surface energy of the coating decreases due to the increase in uncoated PDMS particles, which influence the hydrophobic equilibrium of the coating surface and affect the surface superhydrophobicity.

Accordingly, an appropriate surface roughness is indispensable for the preparation of superhydrophobic coatings. It was found that the technological parameters for obtaining optimal superhydrophobicity (160° WCA, 3° SA) on the coating surface were 0.02 g CNFs, 0.07 g CeO_2_, and 0.3 g PTFE. As shown in Figure 2d, the surface resistivity of the coating decreased gradually with increasing CNF content due to the excellent conductivity of the CNFs. Table S1 (Supplementary Materials) shows the surface resistivity and logarithmic values of the superhydrophobic coating with different carbon contents (g and wt%). At 0.02 g CNFs (2.22%), the surface resistivity decreased sharply to 3.10 × 10^3^ Ω, and the variation of surface resistivity tended to be stable with the increase in CNF content. This phenomenon can be explained by percolation theory. As the CNF content increases, the continuous conductive paths gradually form in the coating, leading to a continuous decline in the surface resistivity. As the CNF content exceeds 0.03 g, the coating percolation ratio is close to 100%, resulting in the growth rate of the percolation group no longer varying significantly with increasing CNF content. It is clear from the above analyses that the coating surface not only possesses excellent superhydrophobicity, but also fine conductivity at 0.02 g CNFs. Therefore, the optimal technological parameters of the CSC coating can be determined to be 0.02 g CNFs, 0.07 g CeO_2_, 0.3 g PTFE, 0.4 g PPS, and 0.1 g PDMS.

### 3.2. Surface Morphology and Wettability

**Figure 3:**
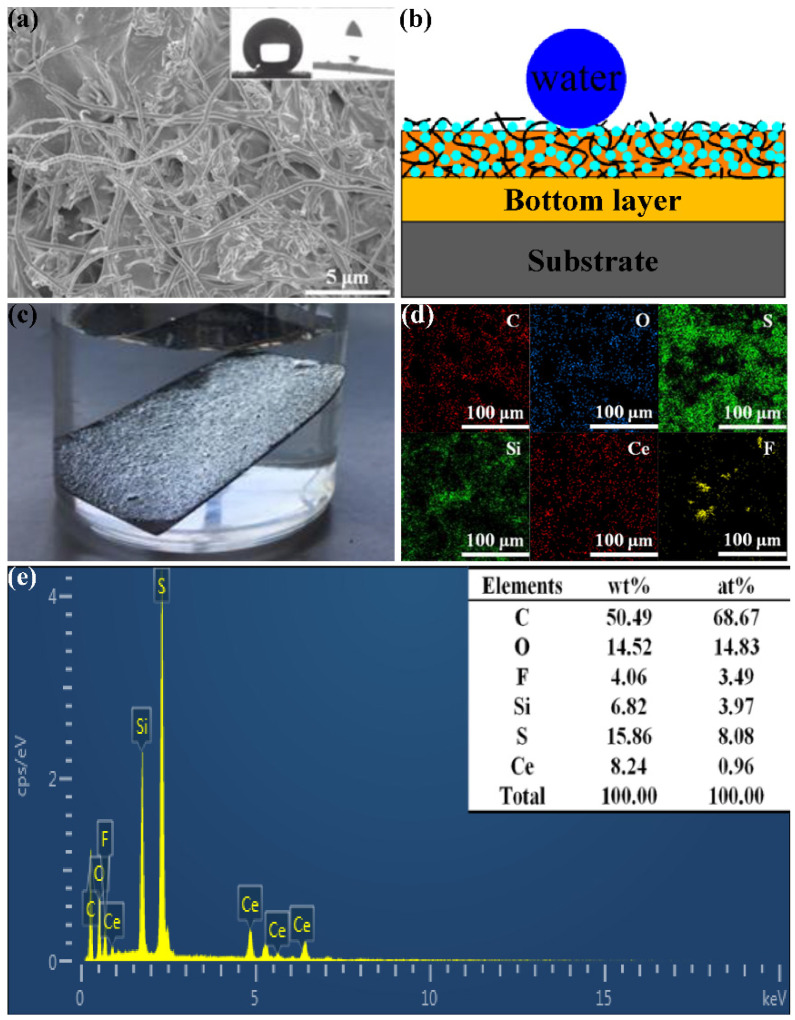
(**a**) SEM images of the CSC coating (insets are the WCA and SA). (**b**) Schematic diagram of superhydrophobicity on the CSC coating surface. (**c**) Silver mirror effect of the CSC coating. (**d**) Element distribution on the CSC coating surface. (**e**) The EDS spectrum of the prepared CSC coating [33]. Reprinted from Ref. [33].

The surface micromorphology of the optimized CSC coating is shown in Figure 3a, and the insets are the WCA (160°) and SA (3°). The coating surface consists of various cured additives and displays the interlaced rough microstructure, which is of vital importance to the superhydrophobicity of the CSC coating. Figure 3c shows the silver mirror effect due to the CSC coating surface superhydrophobicity. According to Cassie theory [35], the air in the concave convex micro-nano structure reduces the contact area between water droplets and solid surface, improving the surface hydrophobicity. The superhydrophobicity of the coating mainly depends on the synergistic effect of surface energy and micro-nano structure. The corresponding element distribution on the CSC coating surface is displayed in Figure 3d, and it can be seen that elements of various additives are distributed on the coating surface. According to our previous work [33], the EDS spectrum of the obtained CSC coating surface is shown in Figure 3e and it can be easily seen that the CSC coating is composed of C, O, F, Si, Ce, and S. Additionally, the peaks of the F, Si, Ce, and S were attributed to the presence of PTFE, PDMS, CeO_2_, and PPS in the MSC coating, respectively. Figure 3b exhibits the corresponding schematic diagram of superhydrophobicity. The interlaced rough microstructure plays an important role in supporting water droplets. Moreover, the superhydrophobicity of the CSC coating depends on the synergistic effect of the rough structure and surface energy of the additives. As the sample is immersed in the liquid, the silver mirror phenomenon appears on the CSC coating surface, which is ascribed to air bubbles filling between the rough structure and the liquid.

Figure S1 (Supplementary Materials) shows the cross-section morphology of the CSC coating. It is easy to see that the total thickness of the coating is about 70 μm, which includes the top layer (30 μm) on the left and the bottom layer (40 μm) on the right. Furthermore, no obvious crack could be found between the top layer and the bottom layer, indicating that the combination between the two layers was very close, which is conducive to improving the bonding performance between the coating and the substrate. 

**Figure 4:**
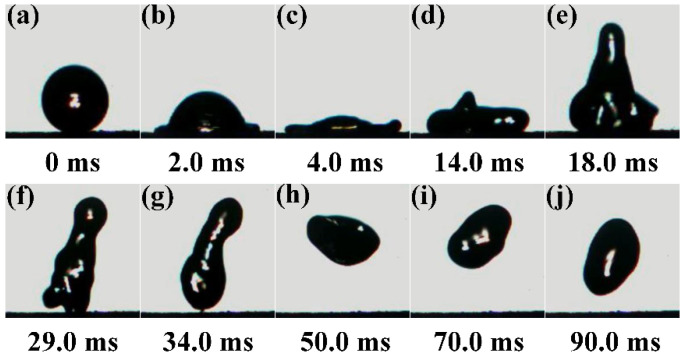
(**a**–**j**) The process of impacting, spreading, and bouncing of water droplets on the CSC coating surface.

The process of impacting, spreading, and bouncing of water droplets on the CSC coating surface is displayed in Figure 4. After 2.0 ms, obvious cap shape and cake deformation of the water droplet was observed. After 14.0 ms, the droplet began to contract and rebound, and the water droplet completely bounced off the surface at 34.0 ms, leaving no residual droplet. The above experimental phenomenon shows that the surface energy of the CSC coating is low, and the adhesion of the coating to the water drop is small. The calculation process of coating surface energy is shown in the supplementary material (Table S2 and Formula (1)–(4)) and the results show that the CSC coating surface energy was about 20.7 mJ/m^2^, which is helpful to realize the superhydrophobic property of the CSC coating. 

### 3.3. Self-Cleaning and Anti-Fouling Properties

**Figure 5:**
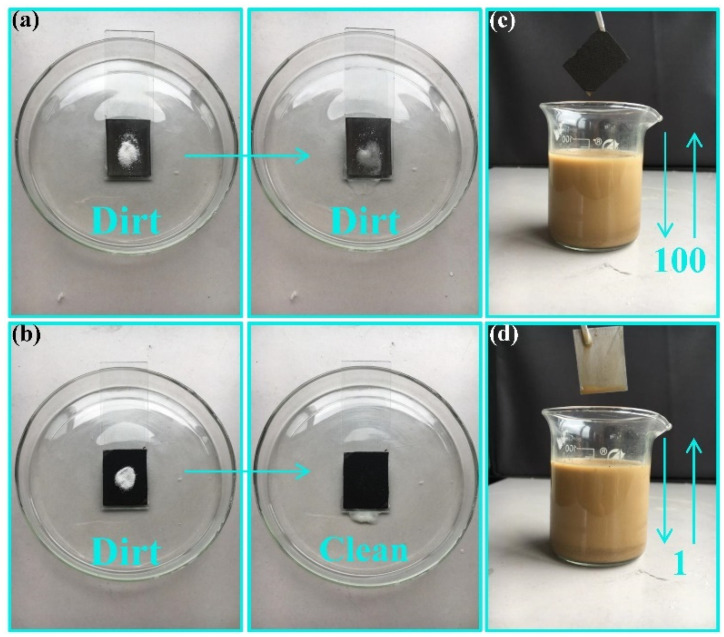
Self-cleaning property of the original sample (**a**) and CSC coating (**b**). Anti-fouling property of the CSC coating (**c**) and original sample (**d**).

In practical applications, the cleanliness of the specimen surface has a great influence on the various properties of the sample [36,37]. Superhydrophobic surfaces have been well applied during the surface cleaning and protection of metal specimens [38,39,40]. In this work, titanium dioxide powders were used as the contaminants. Figure 5a,b shows the self-cleaning properties of untreated and coated specimens, respectively. First, a certain amount of white titanium dioxide powder was dispersed on the surfaces of the two samples. Then, the powder particles on the surface of the sample were washed with water droplets. It is clearly evident that a large number of pollutants still remained on the surface of the original sample, which can be attributed to the high surface adhesion. As water drops fall on the surface due to the high surface adhesion, they flow slowly and do not have enough energy to completely remove the pollutants. 

In contrast, the surface of the coated samples was clean, and no residual contaminant particles were found on it. Therefore, it verified that the surface of the CSC coating possessed excellent self-cleaning property. The low adhesion of the superhydrophobic surface is determined by the surface energy and the micro-nano structure. The self-cleaning property mainly depends on the low adhesion of the coating surface and the adsorption of pollutants by water droplets. As water drops fall on the superhydrophobic surface, with slight drag, spherical water droplets roll fast and have enough energy to remove pollutants. The anti-fouling property is verified by immersing the sample into muddy water made up of a certain amount of water and soil. The sample is immersed vertically in the mud–water mixture, and then the sample is removed from the solution. The above process is recorded as a complete cycle. Figure 5c,d shows the appearance of the coated sample after 100 immersion cycles and the original specimen after one immersion cycle, respectively. From the two pictures, it is easy to see that the surface of the coated sample was not contaminated, and a large quantity of contaminated mixture was left on the surface of the original sample, illustrating the outstanding anti-pollution performance of the CSC coating. This phenomenon is still closely related to the low adhesion of the coating surface. 

**Figure 6:**
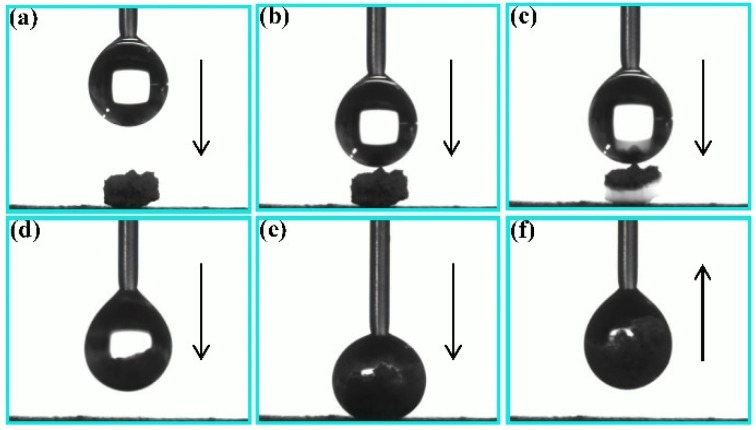
Principle verification of self-cleaning and anti-pollution properties of the CSC coating surface (**a**–**f**).

Extensive research has been used to reveal the self-cleaning and anti-pollution mechanisms of superhydrophobic surfaces, but few experiments have been conducted to verify this hypothesis. Figure 6 displays the process of the corresponding principle verification. In Figure 6a–c, it can be observed that the soil particles are absorbed into water droplets as water droplets fall in contact with soil particles. Afterward, in Figure 6d–f, as the droplets continue to drop and come into close contact with the CSC coating surface, the soil particles, not being separated from the water droplet, remain in the droplet. In addition, after the droplet moves upward to separate from the CSC coating surface, no residual mixture solution was found on the surface, suggesting that the CSC-coating surface has a low liquid adhesion property. Therefore, it can be concluded that the low surface adhesion force and the adsorption of water droplets on contaminated particles are the main factors affecting the self-cleaning and anti-fouling properties of superhydrophobic surfaces. 

### 3.4. Durability Analysis

**Figure 7:**
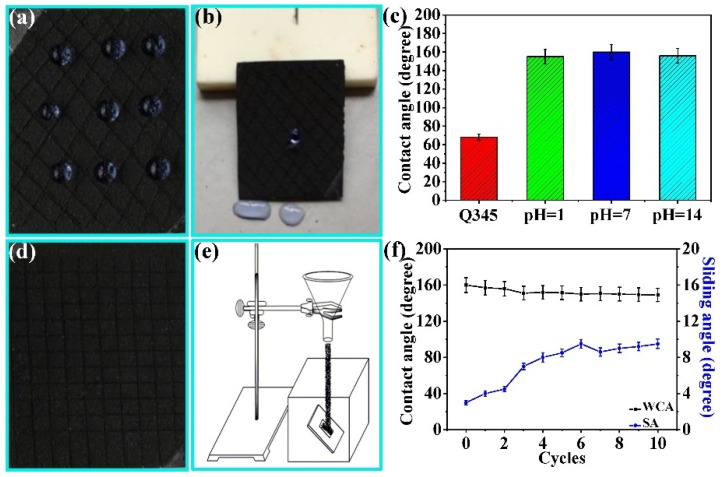
The WCA (**a**) and SA (**b**) of the CSC coating after scraping damage. (**c**) The WCA of Q345 and surface wettability of the CSC coatings after soaking in different pH solutions for 1 h. (**d**) Surface morphology of the CSC coating after the grid test. (**e**) Schematic diagram of the sand-washing device. (**f**) Variations in the WCA and SA of the CSC coating at different sand washing cycles.

Durability is the essential performance metric for superhydrophobic coatings applied in practical production, which is of vital importance to the development of superhydrophobic research [41]. In this paper, a series of experiments were used to verify the durability of the CSC coatings. Figure 7a,b shows the WCA and SA of the CSC coating after scraping damage, respectively. Even though the coating surface is scratched, the water droplets remain spherical and easily roll down from the surface at a low inclination angle, indicating that the damaged coating surface still possesses fine superhydrophobicity, which can be attributed to the good adhesion between the coating and the substrate. The PPS provides excellent adhesion between the coating and the substrate. After being scratched, the surface still retains the micro-nano structures required for superhydrophobicity. In Figure 7c, the WCA of the original sample was 68°. After immersing the coating in solution (pH = 7) for one hour, the WCA of the CSC coating surface was 159°. Even soaking in strong acid (pH = 1) and alkali (pH = 14) solutions for 1 h, the surface of the CSC coating still displayed excellent superhydrophobicity (155° and 156°). The above results illustrate the stable superhydrophobicity of the CSC coating. The low surface energy of the coating is provided by the intrinsic hydrophobic additives (PDMS and PTFE), and no modifier was used. Therefore, the strong acid and alkali had little effect on the surface superhydrophobicity.

In practical engineering applications, coating surfaces are often eroded by gravel, which has a great influence on the coating surface properties [42]. Therefore, it is necessary to simulate a gravel environment in practical applications to evaluate the performance of the coatings. Sand washing, a typical experimental method, is typically used to evaluate the durability of coatings [43]. Based on the above analysis, the sand-washing experiment was adopted to simulate the gravel test in practical applications. A schematic diagram of the homemade sand-washing experimental device is displayed in Figure 7e,f, exhibiting the corresponding experimental results. With the increase in the number of sand-washing cycles, the WCA of the CSC coating surface increased, and the SA showed an upward trend. After 10 sand-washing cycles, the CSC coating surface still retained a stable superhydrophobicity (149° WCA, 9.5° SA). CeO_2_ and PTFE particles had excellent wear resistance. When the sand grains impact the surface, the wear-resistant particles can prevent the surface micro-nano structures from being destroyed. Furthermore, the intrinsically hydrophobic PDMS and PTFE can provide stable low surface energy for superhydrophobicity. Therefore, the CSC coating surface still remains superhydrophobic after multiple sand-washing abrasion. 

### 3.5. Adhesiveness Analysis

**Figure 8:**
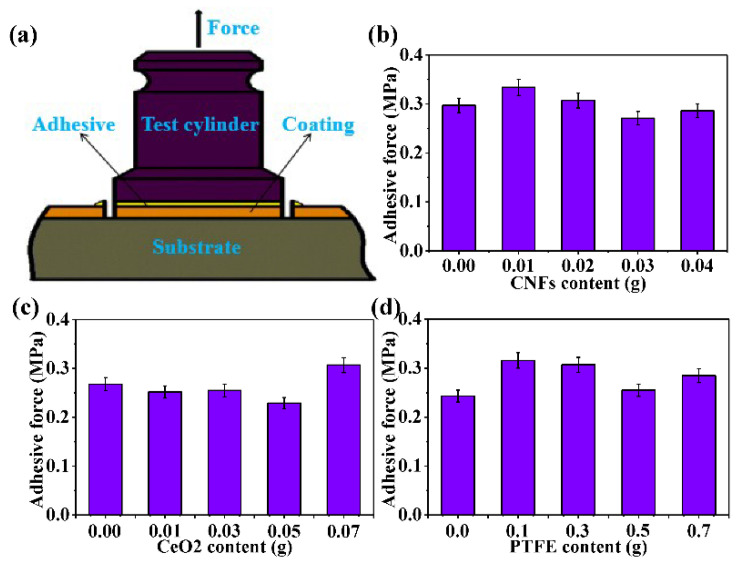
(**a**) Schematic diagram of the pull-off experimental device. Variations in the adhesive force of the CSC coating at different (**b**) CNFs, (**c**) CeO_2_, and (**d**) PTFE contents.

The adhesion between the coating and matrix is the basis of practical engineering applications and various sample properties [44,45]. Consequently, it is necessary to estimate the adhesiveness of the CSC coating and explore the effect of additive content on the adhesion properties of the CSC coating. Here, the grid and pull-off methods were adopted to evaluate the adhesiveness between the coating and the substrate. Figure 7d shows the surface morphology of the CSC coating after the grid test. It is clear that the scratches are smooth and neat, and no obvious shedding of the coating is observed at the crossing, which suggests excellent adhesiveness between the CSC coating and substrate. This result can be attributed to the double-layer spray coating. Additives in the CSC coating have great influence on the adhesion between the PPS and substrate. Spray coating the PPS bottom layer can improve the poor adhesion between the top layer and the substrate. After heat cured, the bottom layer PPS coating can be crosslinked with the top layer PPS based composite coating to enhance the adhesion of the CSC coating. Adhesive force, being the direct and objective factor in evaluating coating adhesion, is measured by the pull-off experimental device, and the schematic diagram is exhibited in Figure 8a. The variations in the adhesive force at different CNFs, CeO_2_, and PTFE contents are displayed in Figure 8b–d. The adhesion force first increases and then decreases with increasing CNF content, which is similar to the change trend of the adhesive force at different PTFE contents. The above variations can be ascribed to the distinct dispersion effect of the spray coating solution at different additive contents. In the initial stage, the spray coating solution dispersed well with increasing additive content, and a tight and uniform coating surface was obtained by spray coating, which is beneficial for enhancing the adhesion force of the coating surface after heating and curing. However, when the additives are further increased, the dispersion of the spray coating solution becomes worse; the surface of the coatings obtained by spray coating is not uniform, and the particles are not compact after heating and curing, which has a great influence on the adhesiveness of the coating and reduces the adhesive force.

In contrast, as the CeO_2_ content was not greater than 0.05 g, the adhesion force decreased with increasing CeO_2_ content, which was ascribed to the poor dispersion of the spray coating solution. However, at 0.07 g CeO_2_, the adhesion force suddenly rose to its maximum due to the improvement in the spray coating effect. In detail, a small amount of CeO_2_ particles can affect the dispersion and spray coating effect of coatings because of their own insolubility. The inhomogeneous distribution of particles in the coating results in poor cohesion, affecting the adhesion of the coating. Furthermore, the cohesion decreases with increasing CeO_2_ particle content. In addition, when the oxide particles increase to a certain amount, the uniformity of oxide particles sprayed in the coating is improved, and the cohesion of the coating is increased, which leads to an increase in the adhesion of the coating. In Figure 8b–d, it can be easily seen that the process parameters with the greatest adhesion force (0.334 MPa) were 0.01 g CNFs, 0.07 g CeO_2_, and 0.3 g PTFE. Moreover, in Figure 2a,d, both the superhydrophobicity (146° WCA, 11.5° SA) and conductivity (2.16 × 10^6^ Ω) of the composite coatings were not ideal. In Figure 8b, the adhesion force of the CSC-coating was 0.307 MPa (<0.334 MPa) at 0.02 g CNFs, but the coating possessed better superhydrophobicity and conductivity than that at 0.01 g CNFs. Therefore, it can be concluded that the optimum adhesion force of the coating is 0.307 MPa.

### 3.6. Wearability Analysis

**Figure 9:**
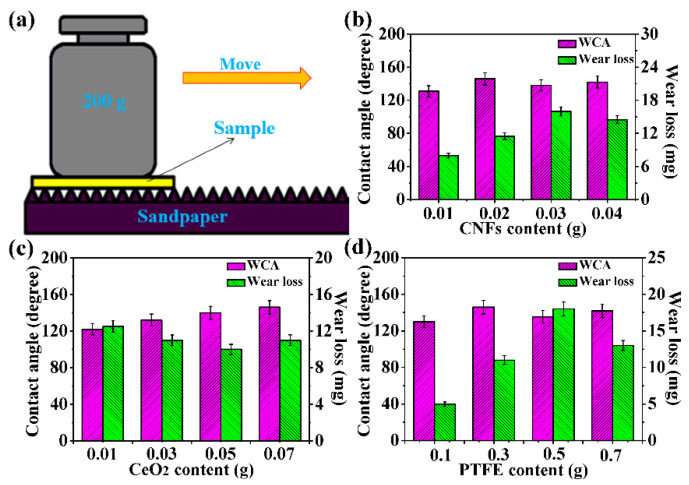
(**a**) Schematic illustration of the abrasion test. Variations in the WCA and wear loss of the CSC-coating at different (**b**) CNFs, (**c**) CeO_2_, and (**d**) PTFE contents after abrasion.

Wear resistance has become a bottleneck restricting the practical application of superhydrophobic materials, and it is also an important issue for current scientific research in this field [46,47]. A simple simulated wear test was adopted to evaluate the mechanical stability of the coating, and a schematic illustration of the abrasion test is displayed in Figure 9a. In addition, the variations in the WCA and wear loss at different CNFs, CeO_2_, and PTFE contents are exhibited in Figure 9b–d. In Figure 9b, it is easy to see that the WCA increased first and then decreased with increasing CNFs content and decreased to varying degrees compared with that before abrasion, which was attributed to the destruction of the coating surface microstructure after abrasion. Simultaneously, the wear loss increased first and then decreased with increasing CNFs content. The above phenomena can be explained by the fact that coating roughness increases with increasing CNF content and that increasing coating roughness influences the coating wear resistance. Moreover, the wear loss decreased when the CNFs content exceeded 0.03 g, which may be related to the friction reduction property of the CNFs acting as lubricants during the wear process. In Figure 9d, the variations in the WCA and wear loss were similar to those at different CNF contents. The wear loss decreased at 0.7 g PTFE, which can be ascribed to the excellent abrasiveness. At different CeO_2_ contents, the WCA increased with increasing CeO_2_ content, which was attributed to the increase in the surface roughness of the coating. However, due to its fine wear resistance, the wear loss decreased with increasing CeO_2_ content as the CeO_2_ content was less than 0.05 mg. At 0.07 mg CeO_2_, the wear loss rose due to the increasing surface roughness.

**Figure 10:**
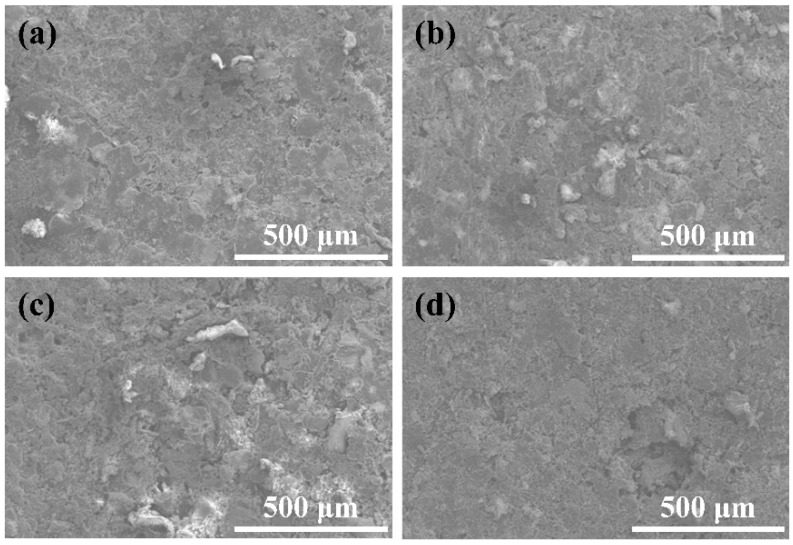
Abrasion morphology of the CSC-coating with (**a**) 0.01 g, (**b**) 0.02 g, (**c**) 0.03 g, and (**d**) 0.04 g CNF contents.

**Figure 11:**
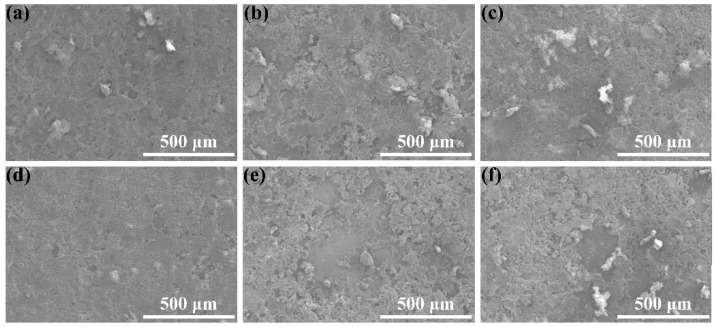
Abrasion morphology of the CSC coating at different CeO_2_ ((**a**) 0.01 g, (**b**) 0.03 g, (**c**) 0.05 g) and PTFE ((**d**) 0.1 g, (**e**) 0.5 g, (**f**) 0.7 g) contents.

The corresponding abrasion morphologies are exhibited in Figure 10 and Figure 11. In Figure 10a, after the abrasion, obvious scaly abrasion marks were found on the surface. In Figure 10b,c, the abrasion on the coating surface became worse with increasing CNF content. Meanwhile, the abrasive surface began to exhibit protruding granular wear marks. However, at 0.04 g CNFs, partial abscission could be clearly seen on the coating surface in Figure 10d. The above phenomena may be explained by the fact that increasing the carbon nanofiber content can reduce the cohesion within the coating. 

The abrasion morphology of the coating at different CeO_2_ and PTFE contents is displayed in Figure 11. At different CeO_2_ contents, scaly and granular wear marks appeared on all wear surfaces (Figure 10b and Figure 11a–c) as well as no obvious coating peeling. This indicates that increasing the CeO_2_ particulate content does not aggravate the abrasion of the coating, which is attributed to the excellent wear resistance of the CeO_2_ particulate. In Figure 11d, the surface of the coating was slightly punctuated with abrasion marks. At 0.3 g PTFE, compared with Figure 11d, the coating abrasion was aggravated and many clear granular wear marks appeared on the coating surface in Figure 10b. Furthermore, the surface wear of the layer was further aggravated with the continuous increase in PTFE content, and a large area of coating peeling occurred in Figure 11e,f. It can be proven that the antifriction property of PTFE particles is beneficial in reducing the abrasion of the coating as the PTFE content is low. However, as the PTFE content increased, the cohesion of the coating decreased, which led to the worsening of coating abrasion. 

**Figure 12:**
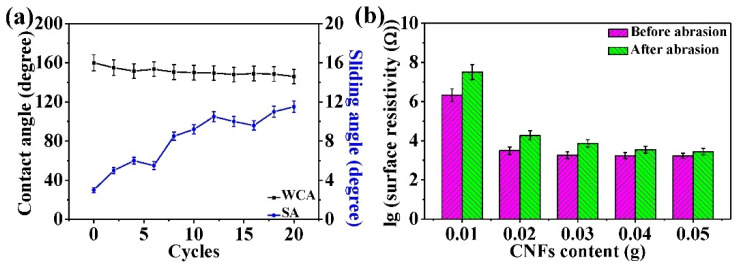
(**a**) Line charts of the WCA and SA of the optimized CSC coating at different abrasion cycles. (**b**) Variations in the surface resistivity of the CSC coating at different CNF contents before and after abrasion.

In Figure 12a, due to the microstructure damage of the superhydrophobic surface, the superhydrophobicity of the optimized CSC coating surface decreased gradually with increasing abrasion cycles. After 10 abrasion cycles, the surface of the optimized CSC coating still maintained fine superhydrophobicity (150° WCA, 9.2° SA) at 200 g pressure. In a previous study [48], the superhydrophobicity of the prepared coating was lost after seven abrasion cycles at 200 g pressure. The above comparison results illustrate that the optimized CSC coating had excellent superhydrophobic stability, which is closely related to the wear resistant and intrinsic hydrophobic additives in the CSC coating as well as the adhesion of the CSC coating. During the abrasion process, the wear resistant additives and the adhesion of the CSC coating ensure that the coating can maintain the required micro-nano rough structure. The intrinsic hydrophobic additives can provide stable low surface energy for superhydrophobicity. The CNF content plays a decisive role in the conductivity of the CSC coating. Therefore, at different CNF contents, it is necessary to research the variations in the coating surface resistivity before and after abrasion. The corresponding experimental results are shown in Figure 12b and Table S3 (Supplementary Materials). At different CNF contents, the variation trends of surface resistivity were similar before and after abrasion. The surface resistivity of the abrasion coatings increased to varying degrees compared with that of the original coatings, which is related to the CNF structural damage on the coating surface. However, at 0.02 g CNF, the surface resistivity (1.86 × 10^4^ Ω) of the optimized CSC coating still met the requirement of an anti-static coating after abrasion, indicating its excellent anti-static stability. This can be explained by the fact that the surface of the CSC coating still maintains continuous conductive paths after multiple abrasion cycles.

## 4. Conclusions

In this work, a stable and durable conductive superhydrophobic coating (160° WCA, 3° SA and 3.10 × 10^3^ Ω) with a facile fabrication process was obtained by a double-layer spray coating method. The optimal technological parameters of the CSC coating were 0.02 g CNFs, 0.07 g CeO_2_, 0.3 g PTFE, 0.4 g PPS, and 0.1 g PDMS. The fabricated superhydrophobic coating displayed excellent self-cleaning and anti-fouling properties. It can be concluded that the low surface adhesion force and the adsorption of water droplets on contaminated particles are the main factors affecting the self-cleaning and anti-fouling properties of superhydrophobic surfaces. The durability is attributed to the abrasion resistant and intrinsically hydrophobic additives making up the CSC coating surface. Even though the surface microstructure is destroyed, the obtained composite coating has stable superhydrophobicity and conductivity. In addition, it also exhibited fine wear resistance and adhesion (0.307 MPa) between the composite coating and substrate. At 200 g pressure, the surface of the obtained CSC coating still maintained fine superhydrophobicity (150° WCA, 9.2° SA) and conductivity (1.86 × 10^4^ Ω) after 10 abrasion cycles. This stable and durable CSC coating, with a facile method and inexpensive preparation process, may be applied in specialty fields to expand the applications of superhydrophobic surfaces. This functional CSC coating can also provide reference value for the preparation and practical application of superhydrophobic surfaces, and may have a broad practical application prospects.

## Data Availability

Data is contained within the article or supplementary material.

## Supplementary Materials

The following are available online at https://www.mdpi.com/article/10.3390/nano11061506/s1, Figure S1: Cross section morphology of the CSC coating, Table S1: The effect of CNFs content on the surface resistivity, Table S2: The contact angle, dispersion force and polarity force of water and Diiodomethane on polished DSC coating surface, Table S3: The effect of CNFs content on the surface resistivity before and after abrasion.
