# Supplementary material for: Stable and Durable Conductive Superhydrophobic Coatings Prepared by Double-Layer Spray Coating Method
Source: Nanomaterials (Basel). 2021 Jun 7;11(6):1506. doi: 10.3390/nano11061506 (PMC8228788; doi:10.3390/nano11061506)
Supplement: Supplementary file 1 [file nanomaterials-11-01506-s001.zip › nanomaterials-1219428-supplementary.pdf]

Supplementary Materials

Stable and Durable Conductive Superhydrophobic Coatings Prepared by Double-Layer Spray Coating Method

Xiang Liu [1], Kai Chen [2], Dekun Zhang [2,*] and Zhiguang Guo [3,4,*]

[1] School of Mechatronic Engineering, China University of Mining and Technology, Xuzhou 221116, China; TB17050017B4@cumt.edu.cn
[2] School of Materials and Physics, China University of Mining and Technology, Xuzhou 221116, China; cumtck@cumt.edu.cn
[3] Hubei Collaborative Innovation Center for Advanced Organic Chemical Materials and Ministry of Education Key Laboratory for the Green Preparation and Application of Functional Materials, Hubei University, Wuhan 430062, China.
[4] State Key Laboratory of Solid Lubrication, Lanzhou Institute of Chemical Physics, Chinese Academy of Sciences, Lanzhou 730000, China.
* Correspondence: dkzhang@cumt.edu.cn (D.Z.); zguo@licp.cas.cn (Z.G.)

Table S1: The effect of CNFs content on the surface resistivity

SDBS-CNFs content (g)	wt%	Surface resistivity (Ω)	lg (surface resistivity)
0.01	1.12	2.16×10^6	6.33
0.02	2.22	3.10×10^3	3.49
0.03	3.30	1.83×10^3	3.26
0.04	4.35	1.75×10^3	3.24
0.05	5.38	1.71×10^3	3.23

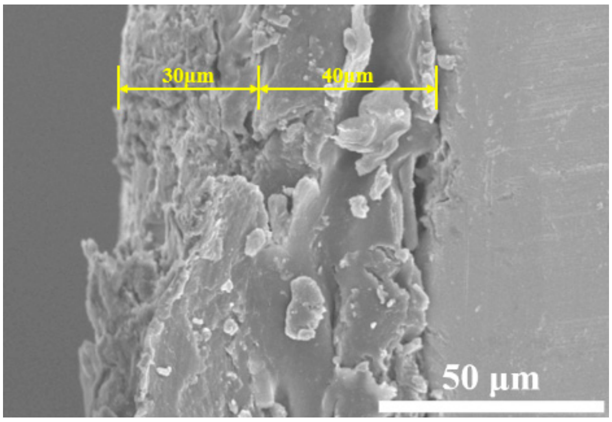

Figure S1: Cross section morphology of the CSC coating

Table S2: The contact angle, dispersion force and polarity force of water and Diiodomethane on polished DSC coating surface

Liquid drop	Contact angle (°)	Dispersion (N/m)	Polar (N/m)
Deionized water	115.3	0.0218	0.0510
Diiodiomethane	75.02	0.0495	0.0013

Surface energy is a significant factor in the fabrication of superhydrophobic surfaces and it can be calculated by the model proposed by Owens [1]. According to the Owens' model, the liquid surface energy can be expressed as follows:

$$\sigma_{lg} = \sigma_l^d + \sigma_l^p \tag{1}$$

where σ_{lg}, σ_l^d and σ_l^p are gas-liquid surface tension, dispersion force and polarity force respectively. The contact angle of liquid on smooth solid surface can be expressed by Young's equation (2).

$$\sigma_{sg} = \sigma_{sl} + \sigma_{lg}\cos\theta \tag{2}$$

where σ_{sg} and σ_{sl} correspond to solid-gas and solid-liquid surface tensions, respectively, and θ is the steady contact angle of smooth solid surface. In this paper, before measuring θ, the superhydrophobic surface should be polished, and then the liquid contact angles were measured on the polished surface. The corresponding liquid contact angles are shown in Table S2. In addition, solid liquid surface energy can be expressed by the following:

$$\sigma_{sl} = \sigma_{sg} + \sigma_{lg} - 2\sqrt{\sigma_s^d \sigma_l^d} - 2\sqrt{\sigma_s^p \sigma_l^p} \tag{3}$$

The relationship between the contact angle and the component forces (equation (4)) can be obtained by simultaneous equations (2) and (3).

$$1 + \cos\theta = \frac{2\sqrt{\sigma_s^d \sigma_l^d}}{\sigma_l} + \frac{2\sqrt{\sigma_s^p \sigma_l^p}}{\sigma_l} \tag{4}$$

The dispersion force and polarity force of water and Diiodomethane are displayed in Table S2. After calculation, we can get $\sigma_{sg} = \sigma_s^d + \sigma_s^p = 20.7$ mJ/m^2, which is the surface energy of the CSC coating.

Table S3: The effect of CNFs content on the surface resistivity before and after abrasion

SDBS-CNFs content (g)	wt%	Surface resistivity (Ω)	lg (surface resistivity)
0.01	1.12	3.24×10^7	7.51
0.02	2.22	1.86×10^4	4.27
0.03	3.30	7.32×10^3	3.86
0.04	4.35	3.50×10^3	3.54
0.05	5.38	2.74×10^3	3.44

References

[1] Owens, D.; Wendt R. Estimation of the surface free energy of polymers. J. Appl. Polym. Sci. 1969, 13, 1741-1747.
